# Soil bacterial community in a photovoltaic system adopted different survival strategies to cope with small-scale light stress under different vegetation restoration modes

**DOI:** 10.3389/fmicb.2024.1365234

**Published:** 2024-03-14

**Authors:** Zhongxin Luo, Jiufu Luo, Sainan Wu, Xiaolin Luo, Xin Sui

**Affiliations:** ^1^China Institute of Water Resources and Hydropower Research, Beijing, China; ^2^National Research Center for Sustainable Hydropower Development, Beijing, China

**Keywords:** photovoltaic, small-scale light stress, soil bacterial communities, vegetation restoration, survival strategies, semi-arid vulnerable areas

## Abstract

Solar photovoltaic (PV) power generation is a major carbon reduction technology that is rapidly developing worldwide. However, the impact of PV plant construction on subsurface microecosystems is currently understudied. We conducted a systematic investigation into the effects of small-scale light stress caused by shading of PV panels and sampling depth on the composition, diversity, survival strategy, and key driving factors of soil bacterial communities (SBCs) under two vegetation restoration modes, i.e., *Euryops pectinatus* (*EP*) and *Loropetalum chinense* var. *rubrum* (*LC*). The study revealed that light stress had a greater impact on rare species with relative abundances below 0.01% than on high-abundance species, regardless of the vegetation restoration pattern. Additionally, PV shadowing increased SBCs’ biomass by 20–30% but had varying negative effects on the numbers of Operational Taxonomic Unit (OTU), Shannon diversity, abundance-based coverage estimator (ACE), and Chao1 richness index. Co-occurrence and correlation network analysis revealed that symbiotic relationships dominated the key SBCs in the *LC* sample plots, with Chloroflexi and Actinobacteriota being the most ecologically important. In contrast, competitive relationships were significantly increased in the *EP* sample plots, with Actinobacteriota having the most ecological importance. In the EP sample plot, SBCs were found to be more tightly linked and had more stable ecological networks. This suggests that EP is more conducive to the stability and health of underground ecosystems in vulnerable areas when compared with LC. These findings offer new insights into the effects of small-scale light stress on subsurface microorganisms under different vegetation restoration patterns. Moreover, they may provide a reference for optimizing ecological restoration patterns in fragile areas.

## 1 Introduction

The atmospheric level of the main greenhouse gas (CO_2_) has reached new record highs in 2021. It has increased by more than 49% from pre-industrial levels (278.3 ppb) to 415.7 ppb, primarily due to emissions from the combustion of fossil fuels and cement production, according to the Global Greenhouse Gas Bulletin from the World Meteorological Organization (WMO). Carbon neutrality is becoming a global consensus for green development (He et al., 2023). Renewable energy is a crucial strategy for reducing CO_2_ emission over time (Chen et al., 2019). Solar energy is a clean and renewable energy source with numerous advantages, including zero carbon emissions, no liquid or solid waste, and widespread availability (Liu et al., 2019). Therefore, solar photovoltaic power (SPP) generation technology is rapidly becoming one of the major carbon reduction technologies (Choi et al., 2020).

Global installed solar photovoltaic (PV) capacity has rapidly expanded, reaching 843.086 GW in 2021. According to the International Energy Agency, global installed solar power capacity is expected to approach 1,700 GW by 2030 (He et al., 2023). However, land constraints will be a major limitation to PV expansion. Arid and semi-arid regions are vast and rich in solar energy resources, making them ideal for PV application. Numerous SPP stations have been constructed in these areas due to the rapid expansion of the PV industry (Liu et al., 2019).

The deployment of large-scale SPP will have a significant impact on the local ecosystem by affecting environmental factors such as the temperature, photosynthetically active radiation, precipitation, evaporation, wind speed, surface albedo, soil heat flux, humidity and temperature, etc. (Broadbent et al., 2019; Yue et al., 2021a). Solar panels partially shading have been shown to delay bloom and increase floral abundance for pollinators in a dryland, agri-voltaic ecosystem (Graham et al., 2021). Nonetheless, most studies have focused on the impact of PV on above-ground macro-ecosystems, and the impact of PV on below-ground micro-ecosystems has not been well studied.

Bacteria are the most prevalent type of soil microorganism and play a crucial role in the material cycling and energy flow. Due to their abundance and high reproductive capacity, bacteria are frequently used as sensitive indicators to evaluate changes in soil ecosystems and characterize soil quality (Trivedi et al., 2016). Light can affect soil temperature, moisture, and nutrient cycling, thus leading to environmental heterogeneity that can impact the composition, distribution, and functional features of soil bacterial communities (SBCs) (Del Pino et al., 2015; Helbach et al., 2022). However, investigations into the effects of light on soil microbial communities have primarily focused on large-scale circumstances, such as the impact of light differences caused by latitude gradients on soil microorganisms (Maestre et al., 2015; Ochoa-Hueso et al., 2018). There are only a few studies on how small-scale light heterogeneity caused by PV arrays affects soil microbial communities, particularly in karst regions with very sensitive geology and heavy human disturbance. A study conducted by Wu et al. (2016) investigated the impact of fluoride and chloride pollution on microbial communities in soils surrounding a solar PV facility. The results indicated a strong correlation between the population size and total biological activity of the SBCs and different levels of fluoride and chloride pollution. The analysis of microbial communities between and under various types of PV panels at Gonghe PV power station, Qinghai Province, has allowed researchers to examine the community abundance, diversity, structure, and distribution characteristics of soil bacteria and archaea. The conclusion drawn from this analysis is that PV stations have minimal impact on the community structure of soil bacteria and archaea (Wu C. et al., 2022; Wu W. et al., 2022; Yuan et al., 2022). However, none of the previous studies have examined the response of soil microbial communities to small-scale light gradients caused by shading from PV modules under different vegetation restorations, nor have they investigated the vertical distribution of bacterial communities. The impact of light changes on soil microbial communities may vary depending on the land use type and vegetation restoration measures employed. This is due to variations in the structure, richness, and diversity of soil microbial communities (Qiang et al., 2015; Lu et al., 2022). Deeper soil microbes play a crucial role in soil formation, nutrient cycling, and carbon storage capacity (Li et al., 2022). However, it is currently unknown how microbial taxa respond to varying soil depths and vegetation restoration in the PV field located in the karst areas of southwest China.

In view of this, this study systematically investigated the influence of light heterogeneity caused by PV panels shading on the diversity and composition of SBCs at different depths (0–20 cm, 20–40 cm, and 40–60 cm) under two vegetation restoration patterns, i.e., *Euryops pectinatus* (*EP*) and *Loropetalum chinense* var. *rubrum* (*LC*), and explored the survival strategies and key driving factors of SBC. The objectives of this study were: (1) to compare the response of SBCs’ composition and diversity to small-scale light stress under different vegetation restoration patterns; (2) to explore the network relationship and survival strategy of SBCs at different depths in the sample plots under different vegetation restoration patterns; and (3) to analyze the key driving factors of the SBCs through correlation network analysis and the Mantel test. This research aims to provide new insights into the effects of small-scale light gradient variation on microorganisms and provide a reference for ecological restoration models in vulnerable areas.

## 2 Materials and methods

### 2.1 Study sites

This study is carried out at the PV Demonstration Base (103° 8′ 30.56″ E, 26° 9′ 55.70″ N) in Dongchuan District (Yunnan, southwest of China) in the semi-arid, vulnerable areas. It has an altitude of about 1,280 m, is in the subtropical monsoon climate zone, and has a characteristic karst terrain. The average annual temperature, rainfall, and evaporation are 14.9°C, 1000.5 mm, and 1856.4 mm, respectively. This zone has a distinct rainy season (from May to September) and a dry season (from October to April). Additionally, the area is rich in solar energy resources, with an annual sunshine duration of over 2,300 h and an annual solar radiation of over 5,000 MJ/m^2^. This PV Demonstration Base covers an area of approximately 18,000 m^2^ with an installed capacity of 1.04 MW. It was officially commissioned in 2020. For the PV arrays, the highest point of the front eaves and rear eaves is 2.5 m and 4.5 m above the ground, respectively. The width of each row of PV panels is 4.2 m, with a tilt angle of 28°and a distance of 2.6 m between two rows. The soil type at the SPP site is predominantly red soil. During the construction process, the soil in the SPP was artificially leveled. After construction, the affected areas were replanted with native plants to mitigate the degradation of the fragile ecosystem.

### 2.2 Sample collection and pretreatment

**Figure 1:**
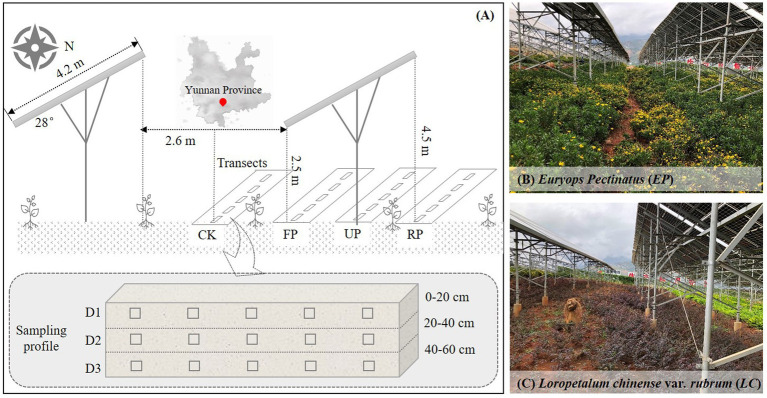
**(A)** Schematic diagram of experimental sampling position in the study area, CK: intervals of PV panels, FP: front of panel eaves, RP: rear of panel eaves, UP: under neath of PV panels; **(B)** *Euryops pectinatus* (*EP*) sample plot; **(C)** *Loropetalum chinense* var. *rubrum* (*LC*) sample plot.

To account for the effects of PV shading on SBCs, the space between two rows of PV panels was considered the control area (CK), and the spaces in the front eaves, back eaves, and under the PV panels were referred to as FP, RP, and UP, respectively. Soil samples were taken from two artificial vegetation plots, i.e., *EP* and *LC*, in October 2021. The litter layer was discarded, and soil samples were collected randomly at the 0–20 cm, 20–40 cm, and 40–60 cm levels from five subplots in each line. The three soil layers, from top to bottom, denoted as D1, D2, and D3, respectively (Figure 1). All the samples were collected at five random sampling sites with three replicates for each location and layer. Samples from the same layer and location in each vegetation plot were mixed and sieved using a 2 mm mesh sieve. Each soil sample was divided into two subsamples: one was air-dried and used for physicochemical analyses, while the other was stored and transported at 4°C and used as soon as possible for soil DNA extraction and high-throughput sequencing.

### 2.3 Soil property measurements

In the flat area of the PV field, a light sensor (FM-G2A, China) was used to monitor the illumination intensity of the top layer of vegetation in 4 locations between and under the PV modules, and temperature and humidity sensors (FM-3A, China) buried at different depths were used to monitor soil temperature and humidity. The heat flux probe (FM-R5, China) is used to monitor changes in soil heat flux in the surface layer of the soil. The monitoring period was the whole month of October 2021, and the monitoring frequency was 30 min each time.

The soil properties, such as soil humidity, pH, electrical conductivity (EC), total nitrogen (TN), total carbon (TC), microbial biomass carbon (MBC), water-soluble organic carbon (WSOC), available phosphorus (AP), and available potassium (AK), are tested with reference to the corresponding standards or previous studies (Lu et al., 2014; Xiao et al., 2017; Pang et al., 2018).

### 2.4 DNA extraction and high-throughput sequencing

Total soil DNA was extracted using a Fast^®^DNA SPIN Kit (MP Biomedicals, Santa Ana, CA, United States) and the method described in the instructions. DNA purity was assessed by agarose (1.0%) gel electrophoresis, and DNA concentrations were quantified using a Nanodrop-2000 device (Thermo Scientific, United States). Universal primers 515F (5′-GTGCCAGCMGCCGCGG-3′) and 909R (5′-CCGTCAA TTCMTTTRAGTTT-3′) were used to amplify the V4-V5 hypervariable regions of the 16S rRNA genes by PCR (95°C for 3 min, followed by 35 cycles at 95°C for 30 s, annealing at 55°C for 30 s, and extension at 72°C for 45 s and then a final extension at 72°C for 10 min) (Li et al., 2014). The PCR product was visually confirmed by agarose gel electrophoresis before purification using AMPure XP beads (Beckman Coulter Inc., Brea, CA, United States). After purification, the PCR products were used to construct libraries and sequenced at Major Bio on an Illumina MiSeq platform (Illumina, United States).

Fastp version 0.20.0 was employed to demultiplex the raw 16S rRNA gene sequencing reads, and FLASH version 1.2.7 was utilized to merge them. UPARSE version 7.1^1^ was used to group operational taxonomic units (OTUs) with a 97% similarity criterion and to detect and remove chimeric sequences. RDP Classifier version 2.2 was applied to compare the taxonomy of each OTU representative sequence to the 16S rRNA database (e.g., Silva v138) with a confidence threshold of 0.7 (Li X. et al., 2023). OTUs were eliminated if they did not contain three reads in at least two samples or were not assigned to a bacterial phylum (Yang et al., 2022). Finally, 2,946 OTUs were used for further analysis.

### 2.5 Statistical analysis

One-way ANOVA and Tukey’s test were applied to the results for the soil properties. The *OTU table* package and the *vegan* package in R software (version 3.5.1) were used to calculate the alpha diversity, and the least significant difference (LSD) was employed to compare the variations in diversity between different subgroups. The significance of changes in the structure of SBCs was examined using non-metric multidimensional scaling (NMDS) analysis, the Wilcoxon rank-sum test, and the Kruskal-Wallis H test, all performed using various functions in the *vegan* package. Correlation analyses and Mantel tests were carried out using the *vegan* package and *ggplot2* package in the R software to examine the association between bacterial diversity and abundance and various environmental factors (R Core Team, 2018). Based on the Random Matrix Theory (RMT), network analysis was performed using the molecular ecological network analysis pipeline. Collinear network analysis was used to show the relationships between different ecological groups and to ensure that only OTU sequences that co-occurred in more than 50% of the sample sites were included in the analysis. The collinear network was constructed using edges with statistical significance (*p* < 0.05). Gephi software was used for visual inspection of the collinear network as well as a computational study of the network properties (Liao et al., 2022).

## 3 Results

### 3.1 Variation of microhabitat in the PV field

**Figure 2:**
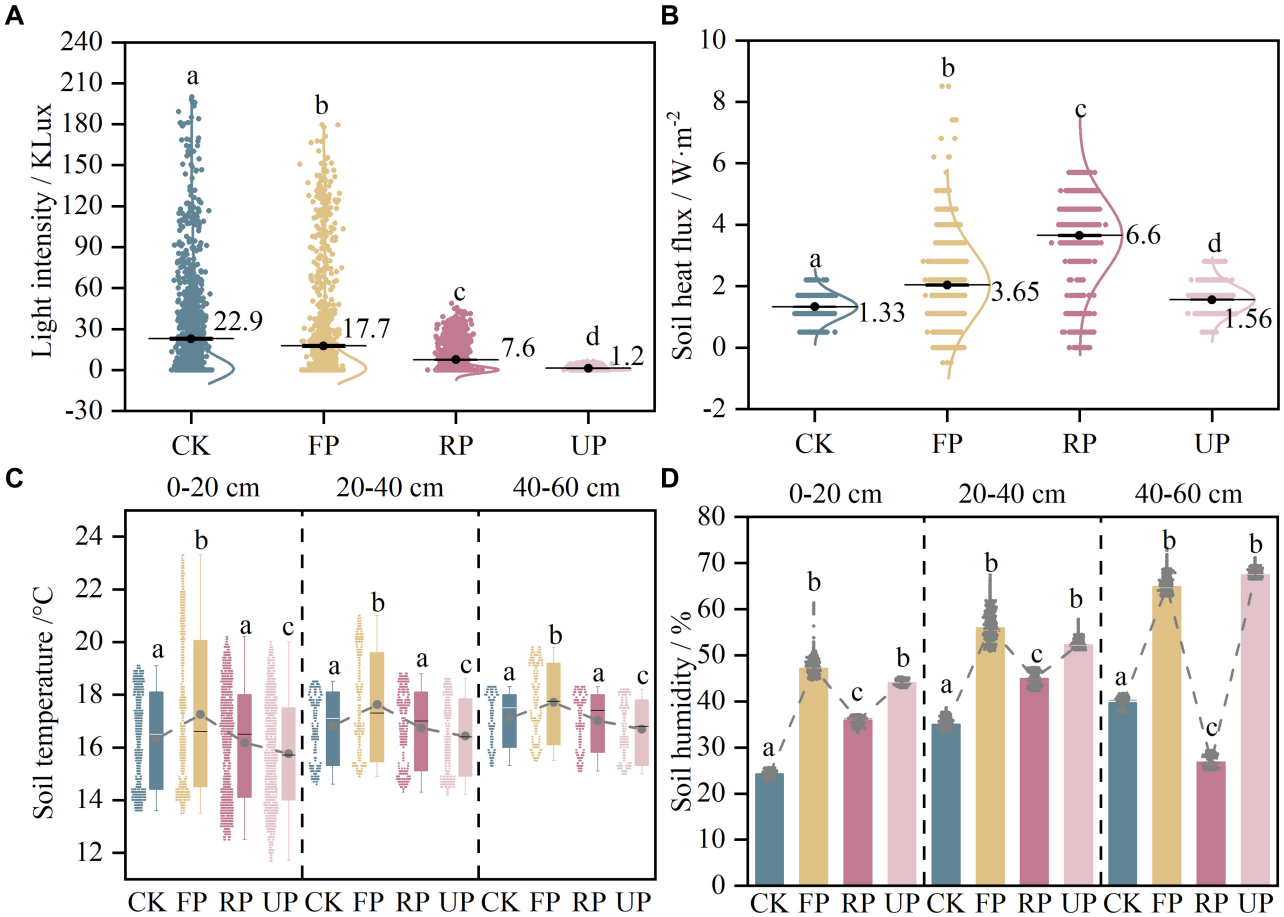
The variation of field monitoring parameters in different positions of the PV field: **(A)** light intensity; **(B)** soil heat flux; **(C)** soil temperature; **(D)** soil humidity. Different lowercase letters indicate significant difference among different location in the PV field (one-way ANOVA, *p* < 0.05).

The light intensity at different locations was markedly different (*p* < 0.05) in the order: CK > FP > RP > UP, indicating that the shading of the PV panels significantly reduced the light intensity and showed a gradient change at different locations (Figure 2A). The soil temperature in different soil layers showed a similar trend, first increasing and then decreasing along the light gradient and being highest at the FP position (Figure 2B). The soil heat fluxes in the shaded area of the PV panels all increased significantly, and the fluctuation range was noticeably larger in FP and RP (Figure 2C). The soil humidity at different depths also showed a similar trend, which followed an N-shaped trend with the light gradient and increased considerably at FP and UP positions (Figure 2D).

The soil properties exhibited varying trends along the light gradient under two vegetation restoration patterns, as shown in Supplementary Table S1. TC, WSOC, pH, EC, AK, and AP were not notably different (*p* > 0.05), while TN, NO_3_^−^-N, MBC, and soil humidity exhibited considerable differences (*p* < 0.05). TN was found to be highest in the RP location of the *EP* sample plots at 1.18 g/kg and was significantly different from the RP and UP locations of the *LC* sample plots. The NO_3_^−^-N content decreased in the *EP* sample site as the light intensity decreased. The difference between the CK and UP sites was considerable. Additionally, it decreased significantly in the RP and UP locations of the *LC* plot (*p* < 0.05). The soil humidity in this study area ranged from 13.53 to 21.25%. The average soil humidity in the shaded area of the PV panels is about 10% higher than the CK position. The variation trend observed was consistent with the long-term monitoring of soil humidity by the meteorological probe. The FP and UP positions showed a pronounced increase. The MBC was lowest at the CK location in both planted sample plots. The PV shadowing boosted SBCs’ biomass by 20–30%, indicating a significant increase in the microbial biomass of the soil due to the PV shading.

### 3.2 Response of SBCs’ composition to environmental heterogeneity

**Figure 3:**
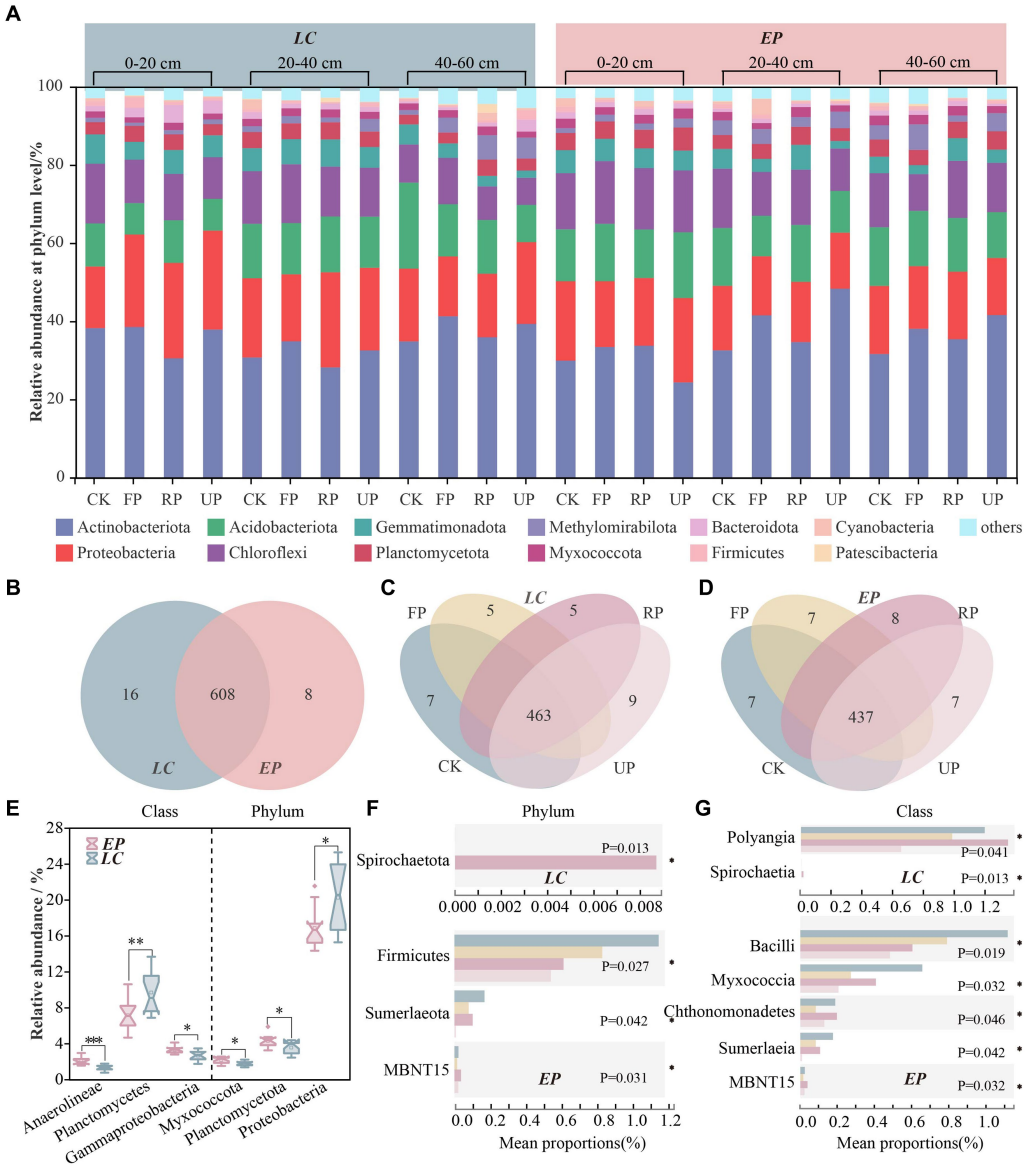
The composition of SBC at the phylum level in two vegetation sample plots **(A)**; Venn diagram at the genus level between *LC* and *EP* sample plots **(B)**; Venn diagrams at genus level along four light gradients of *LC* **(C)** and *EP* **(D)**; A Wilcoxon Rank-Sum Test box plot was used to compare LC and EP plots at the phylum and class level, and only bacteria taxonomic with significant differences are shown **(E)**; A Kruskal-Wallis H test bar plot along light gradient in LC and EP plots at phylum level **(F)** and class level **(G)**.

Following quality filtering, high-throughput sequencing was performed on distinct soil bacterial 16S rRNAs in the PV field, resulting in 979,402 clean reads. The number of genuine high-quality bacterial reads ranged from 32,978 to 47,325, with a corresponding library coverage rate varying from 98.0 to 98.6%. This indicated that the sample libraries in this investigation contained the majority of bacterial taxa and accurately reflected the structural makeup of the bacterial community in the samples. The SBCs comprised 12 prominent phyla, each with a relative abundance of over 2% (Figure 3A). The five most abundant phyla were Actinobacteriota, Proteobacteria, Acidobacteriota, Chloroflexi, and Gemmatimonadota, which accounted for 24.38–48.33%, 11.79–25.31%, 8.05–22.00%, 6.94–17.21%, and 1.85–7.51% of total reads, respectively.

The Venn diagram reveals that 608 genera occurred in both the *LC* and *EP* sample plots, 16 genera were endemic to the *LC* sample plot with relative abundances ranging from 0.027 to 0.564%, and 8 genera were endemic to the *EP* sample plot with relative abundances ranging from 0.004 to 0.244% (Figure 3B). In the LC sample plot, 463 genera co-occurred in different light gradients. The CK had 7 endemic genera, while the FP, RP, and UP had 5, 5, and 9 endemic genera, respectively (Figure 3C). In the *EP* sample plot, 437 genera co-occurred in different light gradients. The CK had 7 endemic genera, while the FP, RP, and UP had 7, 8, and 7 endemic genera, respectively (Figure 3D).

At the phylum level, Proteobacteria, Planctomycetota, and Myxococcota demonstrated notable variations (*p* < 0.05) between the two vegetation sample plots (Supplementary Figure S1). The abundance of Proteobacteria was much higher in the *LC* sample plots than in the EP sample plots, while Planctomycetota and Myxococcota showed the opposite trend. Gammaproteobacteria (*p* < 0.05), Planctomycetes (*p* < 0.01), and Anaerolineae (*p* < 0.001) exhibited significant differences between the two vegetation sample plots. The *LC* sample plots had a significantly higher abundance of Gammaproteobacteria compared to the *EP* sample plots. Conversely, Planctomycetes and Anaerolineae showed the opposite pattern (Figure 3E). The Kruskal-Wallis H test revealed significant differences in the abundance of Proteobacteria and Bacteroidota at varying depths in the *EP* plots, and Chloroflexi, Gemmatimonadota, and Methylomirabilota in the *LC* plots (Supplementary Figure S1). This highlighted that vegetation type can not only influence the composition of the SBCs in the surface layer through differences in litter quality and quantity, but also the distribution of soil bacteria in the deep layer through root exudates and their effects on soil structure.

Figures 3F,G shows Kruskal-Wallis H test bar plots for two sample plots at phylum and class levels along the light gradient. In the *LC* sample plot, Spirochaetota was only present in the RP position, indicating a strict light requirement. Polyangla varied significantly with the light gradient at the class level (*p* < 0.05), while Spirochaetiaj occurred only at the RP position with a relative abundance of less than 0.01%. The *EP* sample plot showed a decrease in the relative abundance of Firmicutes with increasing light gradient. In the PV shade area, Sumeriaeota had a 30 to 90% lower relative abundance compared to CK, while MBNT15 had the highest relative abundance at the RP position. At the class level, Bacilli, Mycococcia, Chthonomonadetes, Sumerlaeia, and norank_MBNT15 revealed substantial variations with light gradients (*p* < 0.05).

### 3.3 Response of alpha and beta diversity of SBCs to environmental heterogeneity

**Figure 4:**
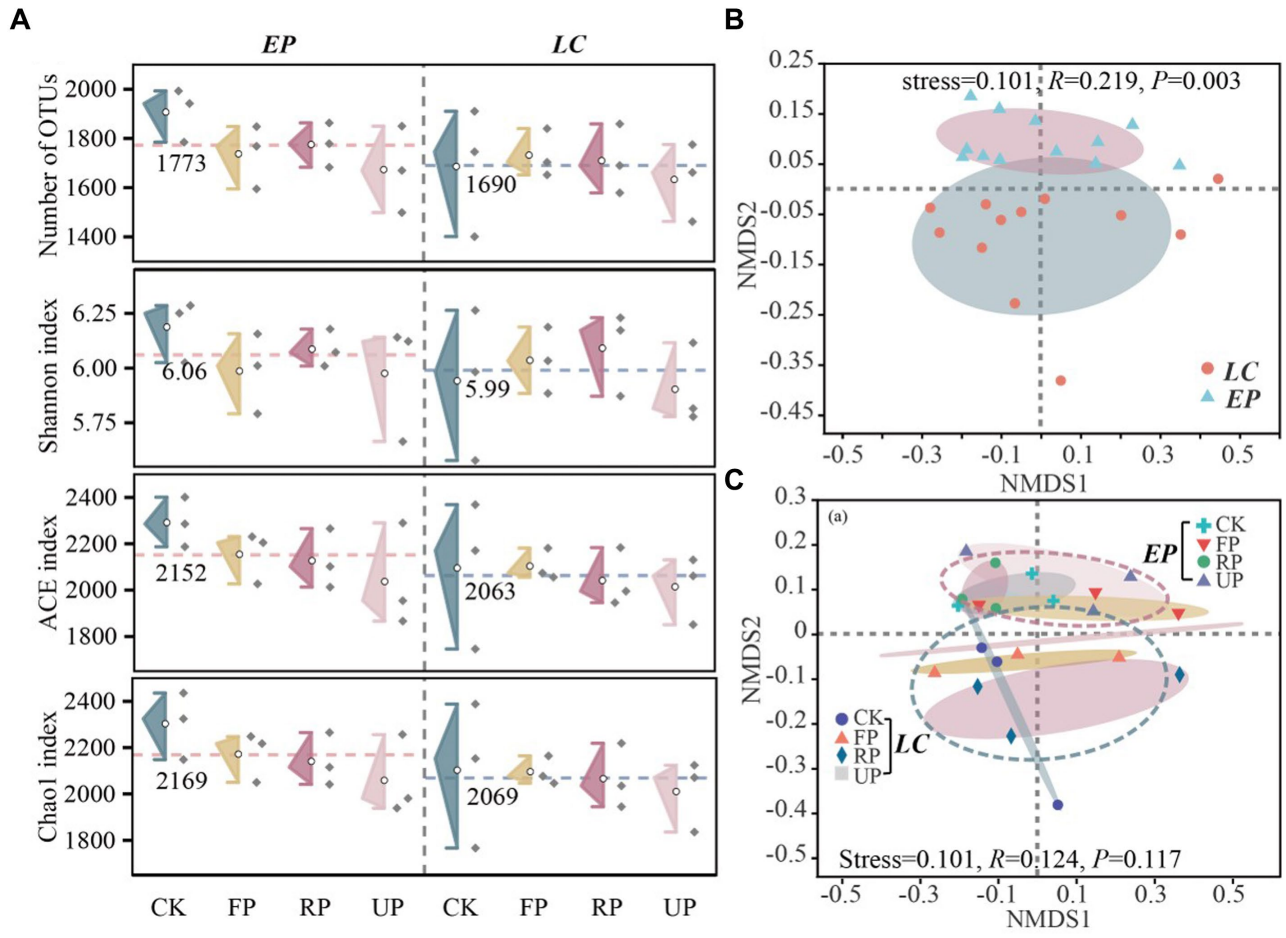
**(A)** Alpha diversity analysis of SBC in different soil location types and layers in two vegetation sample plots. The number of OTUs, Shannon diversity index, ACE evenness index, and Chao1 richness index are shown, with the red and blue dotted lines representing the average diversity index of EP and LC plots, respectively. **(B)** Beta diversity of bacterial community based on Bray-Curtis distance (on OTU level) analyzed by NMDS in two vegetation sample plots, and **(C)** different location along a light gradient.

The OTU numbers, Shannon diversity index, ACE evenness index, and Chao1 richness index were calculated to compare the α-diversity of SBCs. The results revealed inconsistent changing patterns along the light gradient in the LC and EP sample plots (Figure 4A). Although the α*-*diversity index of the *EP* plot was higher than that of the *LC plot*, there was no significant difference (*p* > 0.05). There were no noticeable changes in the numbers of OTU and Shannon diversity index across sampling sites in both *EP* and *LC* sample plots. In the EP sample plots, PV shade reduced both the OTU numbers and Shannon diversity index of SBC compared to CK. However, there was no clear pattern of influence of PV shading in *LC* sample plots. Both the ACE and Chao1 indices decreased gradually as the light gradient decreased, but there was no significant difference between different sampling locations within the same sample plots (*p* > 0.05). It is worth noting that PV shading increased soil microbial biomass by 20–30% (MBC content in Supplementary Table S1), but the OTU numbers, Shannon diversity, ACE and Chao1 richness index decreased to varying degrees.

The bacterial community diversity was reasonably affected by different sampling depths, as shown in Supplementary Figure S2. In the *LC* sample plot, the layer of 20–40 cm layer exhibited the highest bacterial richness and diversity, while in the *EP* sample plot, the bottom layer (40–60 cm) had the highest bacterial richness and diversity. The ACE and Chao1 indices in the layer of 40–60 cm differed significantly between the *LC* and *EP* sample plots (*p* < 0.05). NMDS analysis was conducted on the composition of SBCs at different light gradients in two vegetation plots based on Bray-Curtis distance (Figures 4B,C). The composition of SBCs changed considerably (*p* = 0.003) between the *LC* and *EP* plots, but not significantly (*p* = 0.117) at different light gradients. This indicates that the influence of light intensity on SBC’s composition differed significantly under two vegetation restoration patterns. The bacterial community showed less variability in the layers of 0–20 cm and 20–40 cm compared to the layer of 40–60 cm, particularly in the *LC* sample plot. The vertical distribution of SBCs was most noticeable at the CK site in the *LC* sample plot, while in the *EP* sample plot, the difference was greatest at the UP position. Furthermore, the sample points that represent the composition of SBCs at various locations and depths were more closely clustered in the *EP* sample plot than in the *LC* sample plot.

### 3.4 Comparison of the co-occurrence networks of SBCs

**Figure 5:**
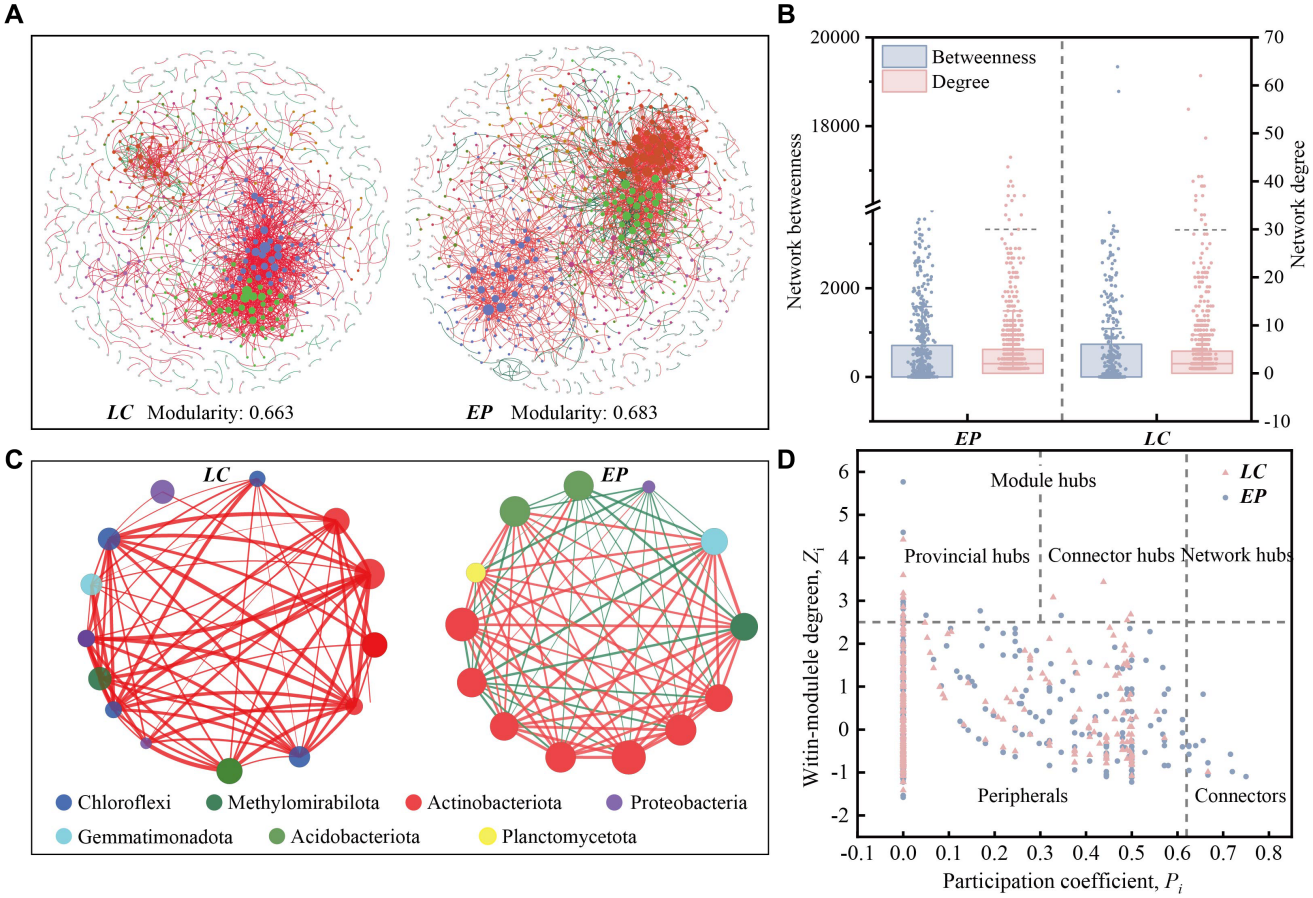
**(A)** Co-occurring network of SBCs at OTU level in the *LC* and *EP* plots. The nodes in network are colored by modularity class. A group of OTUs in one module indicates that these OTUs have more interactions among themselves and fewer associations with other modules. The node sizes reflect their degree of connection; **(B)** Betweenness and node-normalized degree of bacterial networks; **(C)** Network structures of the core genus (Degree >30) in the *LC* and *EP* plots. The nodes in network are colored by phylum. Red edges indicate significant positive Spearman’s correlations, while green edges indicate significant negative Spearman’s correlations; **(D)** The Zi-pi plots of OTUs in bacterial co-occurrence networks are based on topological interactions.

The co-occurrence of SBCs in two vegetation plots was described using the molecular ecological network (MEN) based on random matrix theory (RMT) (Figure 5A). Networks for *LC* (755 nodes connected by 1,744 links) and *EP* were built in various sizes (779 nodes connected with 1,941 links). The created MEN had modular architectures, as evidenced by modularity values higher than 0.4 in both the LC and EP sample plots (Newman, 2003). The integration of one network module’s nodes suggested that the group was tightly knit, with few nodes affiliated with it outside of the module (Williams et al., 2014). The average path distance in the EP plots was smaller, indicating that the nodes were more closely connected to networks (Table 1). However, the *LC* and *EP* plots did not show any discernible difference in betweenness or degree (one-way ANOVA, *p* > 0.05) (Figure 5B).

**Table 1:** Bacterial network properties under two vegetation restoration modes.

Network parameter		*LC*	*EP*
Node	Number of nodes	754	779
	Average degree	4.626	4.983
	Average path distance	6.692	4.978
	Average clustering coefficient	0.305	0.209
	Modularity	0.663	0.683
Edge	Number of edges	1744	1941
	Negative (proportion)	175 (10.0%)	362 (18.7%)
	Positive (proportion)	1,569 (90.0%)	1,579 (81.3%)

In the *LC* sample plot, the core OTUs (degree > 30) were mainly primarily composed of Chloroflexi, Methylomirabilota, Actinobacteriota, Proteobacteria, Gemmatimonadota, and Acidobacteriota. Among these, the ecological niches of Chloroflexi and Actinobacteriota were the most significant, and all species exhibited positive correlations, primarily in symbiotic relationships. In the *EP* sample plot, the core OTUs (degree > 30) were mainly Actinobacteriota, Acidobacteriota, Methylomirabilota, Gemmatimonadota, Planctomycetota, and Proteobacteria. Actinobacteria had the most ecological importance. Planctomycetota and Actinobacteriota became more important in the *EP* plot comparison to the *LC* plot, while Chloroflexi became less important (Figure 5C).

The network nodes were classified into four groups using the fast-greedy approach to determine the primary populations that impact the co-occurrence of SBCs. These groups include network hubs (*Z*_i_ > 2.5, *P*_i_ > 0.62), module hubs (*Z*_i_ > 2.5, *P*_i_ < 0.62), peripherals (*Z*_i_ < 2.5, *P*_i_ < 0.62) and non-hub connectors (*Z*_i_ < 2.5, *P*_i_ > 0.62). The classification was based on the within-module connection (*Z*_i_) and among-module connectivity (*P*_i_). The module hubs were classified as either provincial hubs (*Z*_i_ > 2.5, *P*_i_ < 0.3) or connectors (*Z*_i_ > 2.5, 0.3 < *P*_i_ < 0.62) based on the value of *P*_i_.

The *Z*_i_-*P*_i_ plot reveals that approximately 98.20% of the OTUs were peripheral, implying that most OTUs had fewer linkages outside of their modules. The majority of the peripherals (*P*_i_ = 0) in the *LC* (75.2%) and *EP* (80.9%) plots were exclusively connected within their respective modules. Both the *EP* and *LC* sample plots had 14 module hubs, most of which belonged to Actinobacteriota. In the *EP* sample plot, there was only one connector hub belonging to Proteobacteria, while in the *LC* sample plot, there were three connector hubs, one belonging to Acidobacteriota and two belonging to Actinobacteriota. As keystone taxa, these hubs are crucial for maintaining the structure and functional integrity of the microbial community (Wu et al., 2023). Additionally, there were 14 non-collector connectors in the *EP* sample plot, five of which belonged to Chloroflexi. In comparison, there was only one non-collector connector in the LC sample plots, belonging to Gemmatimonadota (Figure 5D).

### 3.5 Key environmental drivers of SBCs

**Figure 6:**
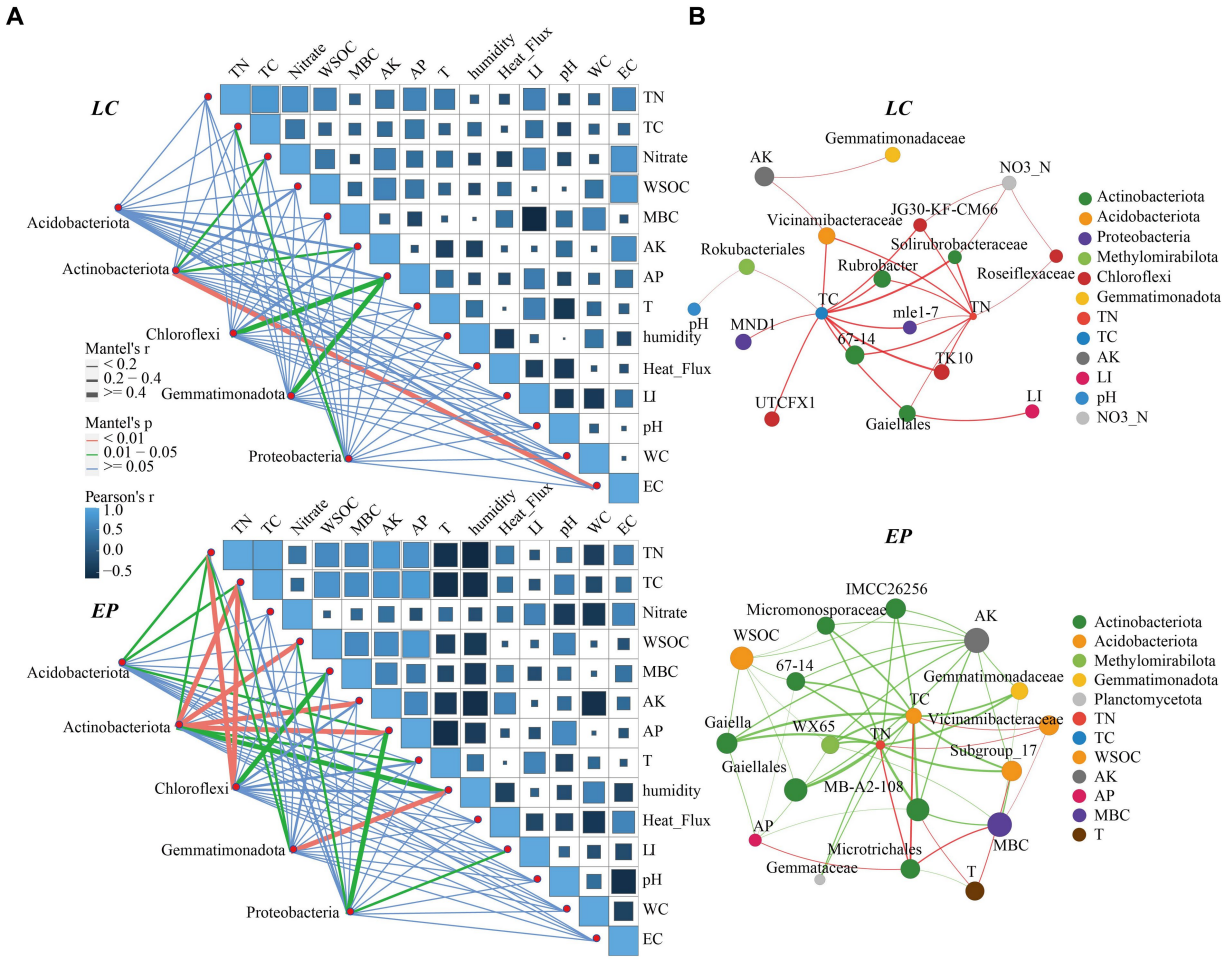
**(A)** Correlation analysis and the Mantel test of soil properties and dominant SBCs in the genus *LC* and *EP* plots; **(B)** Two-factor correlation network analysis of core genera (Degree > 30) and environmental factors in the *EP* and *LC* plots; TC, total carbon; TN, total nitrogen; WSOC, water-soluble organic carbon; MBC, microbial carbon; AK, available potassium; AP, available phosphorus; EC, electric conductivity. LI, Light Intensity.

The Mantel test and correlation analysis were used to examine the relationships between soil properties and dominant SBCs (Figure 6A). The results showed that light intensity had a significant positive correlation with TN, TC, and NO_3_^−^-N, as well as AP, but a negative correlation with MBC in the *LC* sample plot.

Actinobacteriota was significantly correlated with NO_3_^−^-N, AK, and EC; Chloroflexi and Gemmatimonadota were both strongly correlated with AP, and Proteobacteria was highly associated with TC. In the *EP* sample plots, the soil temperature and humidity exhibited a marked negative correlation with soil nutrients. The interaction between dominant bacteria and environmental factors was considerably enhanced, and light intensity was also significantly negatively correlated with MBC. Actinobacteriota showed significant correlation with TC, WSOC, AP, and AK, whereas Chloroflexi was exhibited a strong correlation with TN, TC, and MBC. Collectively, soil nutrients were the main drivers of soil dominant community changes.

Two-factor correlation analysis (Figure 6B) revealed that the core species of soil bacteria had a significant negative correlation with soil nutrients in the *EP* sample plots Conversely, in the *LC* sample plot, they were mainly positively correlated, and light intensity was significantly positively correlated with *Gaiellales*.

## 4 Discussion

### 4.1 Effects of shading of the PV panels on soil properties

The large-scale construction of PV panels can cause heterogeneity in environmental factors, such as light, precipitation, and wind speed. This can lead to microhabitat climate changes that may affect ecosystems (Li C. et al., 2023; Li X. et al., 2023). The study found that light intensity varied significantly (*p* < 0.05) across different locations, with the order being CK > FP > RP > UP. PV panel shielding reduces the amount of solar radiation by limiting the duration and area of direct exposure to the ground. Additionally, fixed-axis PV modules have varying angles, causing changes in the duration and amount of solar radiation at different locations (Marrou et al., 2013; Broadbent et al., 2019; Wu C. et al., 2022; Wu W. et al., 2022). The soil humidity in FP is higher due to the pooling effect of precipitation caused by PV modules (Choi et al., 2020). In the UP position, the shielding effect of PV panels reduces wind speed and solar radiation, increasing air humidity and hindering water evaporation to some extent (Amaducci et al., 2018). Meanwhile, the shading effect of vegetation can effectively reduce surface water evaporation and improve soil water holding capacity (Liu et al., 2019; Yue et al., 2021b).

Soil characteristics are widely acknowledged as crucial indicators of soil fertility and their ability to sustain plant production has been well documented (Zhao et al., 2019). Previous studies have demonstrated that the construction of PV power plants can alter the microclimate environment for vegetation growth by directly or indirectly impacting local airflow, precipitation, solar radiation, air temperature, humidity, and other factors. These changes can indirectly affect soil nutrient conditions (Armstrong et al., 2016; Yue et al., 2021a). Soil humidity is an environmental variable that combines the effects of climate, vegetation, and soil on the dynamics of water-stressed ecosystems. Soil humidity fluctuations can significantly impact plant development in PV fields, especially in dry areas where even small changes in water availability can have major effects on plant growth (D’Odorico et al., 2007). As a consequence, depending on the water requirements of the plants, the environmental heterogeneity provided by PV modules may offer certain advantages (Del Pino et al., 2015). MBC is the active component of the soil organic pool, which is an easily accessible pool of nutrients in the soil and the driving force for organic matter decomposition and nitrogen mineralization, acting as a key source and reservoir in the carbon and nitrogen cycling processes (Lu et al., 2022). Soil MBC is frequently used to estimate the magnitude of soil microbial biomass and is important for soil organic matter and nutrient cycling (Chen et al., 2023). In both vegetation restoration models, shading by PV panels increased the MBC content, i.e., the biomass of microorganisms in the below-ground microbial system. This might be because soil microorganisms in karst areas are prone to high temperature and drought stress, and shading by PV modules decreases soil temperature and increases soil humidity, and then the SBC may thrive and reproduce under optimal temperature and moisture conditions (Tuo et al., 2023).

### 4.2 Effects of shading of PV panels on composition and diversity of SBCs

Bacteria are the most abundant microorganisms in soil and play a crucial role in material cycling, energy flow, and maintaining ecological balance in terrestrial ecosystems. Their activities and interactions are vital for soil fertility, plant health, and ecosystem stability (Zhang et al., 2022; Liu et al., 2023c). The study area revealed five prominent phyla: Actinobacteriota, Proteobacteria, Acidobacteriota, Chloroflexi, and Gemmatimonadota. These findings are consistent with those reported for global drylands, although there are noticeable variations in relative abundance (Ochoa-Hueso et al., 2018).

The relative abundances of SBCs can serve as a biological indicator of environmental health (Lee et al., 2020). The abundance of Proteobacteria, Planctomycetota, and Myxococcota differed significantly between the LC and EP sample plots. This suggests that vegetation restoration regulates SBCs to some extent and that different vegetation types have a significant impact on the dominant microbial community (Liu et al., 2023a). The relative abundance of dominant phylum remained relatively constant across the light gradient in both vegetation plots. However, the sampling depth did vary. This suggests that the impact of light heterogeneity on the soil’s abundant microorganisms was less significant than the soil depth under two different vegetation restoration patterns. The Venn diagram reveals that the endemic genera are relatively less abundant in different positions and vegetation plots, all being less than 1%, indicating that rare species are more responsive to environmental heterogeneity due to higher environmental filtering and dispersal limitations (Luan et al., 2023). These SBCs that produce significant differences under small-scale light heterogeneity, are likely to be photosensitive bacteria, and their functional characteristics deserve in-depth study in the future.

Light has both direct and indirect effects on the abundance and structure of soil microbial communities. Shading of PV panels at PV sites affects the amount of irradiation and energy received by the soil at different locations, which in turn affects the structure of soil microbial communities (Bai et al., 2022). PV shading increased soil microbial biomass by 20–30%. However, the numbers of OTU, Shannon diversity, ACE and Chao1 richness index decreased to varying degrees. This may be due to a decrease in light intensity, which reduced the number of sun-loving microbial species while promoting the growth and reproduction of shade-loving microbial species due to decreased competition (Davies et al., 2013).

### 4.3 SBCs adopt different survival strategies under two vegetation restoration models

In natural ecosystems, microorganisms do not exist as isolated individuals, but form complex networks of co-occurrence through direct or indirect interactions (Fan et al., 2017; Wu et al., 2021). Species interactions are represented by links in co-occurrence networks. Co-colonization, cross-feeding, niche overlap, and co-aggregation in microorganisms lead to positive species correlations, whereas predator–prey associations, misfeeding, improper feeding, and competition lead to negative species correlations (Yang et al., 2021). In the molecular ecological network of the LC plot, 90% of the correlations between species were positive, whereas in the EP plot, the positive correlations decreased significantly to 81.3% (Table 1). Furthermore, the core genera with a degree greater than 30 in the *LC* plot exhibited positive correlations. In contrast, the core genera in the *EP* sample plot showed a significant increase in negative correlations, suggesting an increase in competitive relationships between species and a more stable ecological network. These indicate that the SBCs in the *LC* plot was mainly in symbiotic relationships, while the competitive relationship between species increased and the ecological network became more stable in the *EP* plot (Liao et al., 2022; Liu et al., 2023b). That is to say, SBCs adopt different survival strategies to cope with small-scale light stress under two vegetation restoration modes.

Non-hub connections are responsible for managing the flow of information between modules that are otherwise inadequately or not at all linked to one another. Therefore, deleting non-hub connector species may significantly impact the functional connection of various network modules (Guimera and Nunes, 2005). The higher number of non-collector connectors in the *EP* sample plots, compared to the *LC* sample plots, suggests that the functions of SBCs in the *EP* sample plots were more closely linked and the ecological network was more stable (Liao et al., 2022). This could facilitate better adaptation to the small-scale light stress in the PV site area.

### 4.4 Factors driving SBCs under two vegetation restoration models

The construction and operation of SPP can promote the development of biological soil crust and vegetation growth, leading to an improvement in soil texture and nutrition (Luo et al., 2023). The study found that the relationships between soil properties, light intensity, and dominant bacterial communities varied between the two modes of vegetation restoration. In the *LC* sample plot, light intensity had a significant positive correlation with TN, TC, and NO_3_^−^-N, as well as AP, but a negative correlation with MBC in the *LC* sample plot. These findings suggest that PV panel shading not only facilitates the accumulation of soil nutrients but also the increase of soil microorganisms. The *EP* sample plot showed a significant positive correlation between light intensity and nitrate-nitrogen and a negative correlation with MBC. The interaction between dominant bacteria and environmental factors was also significantly enhanced. This was attributed to differences in plant inter-root secretions and the nutrient cycling of plant litter in different vegetation types, which affect the soil environment and further influence the structure and diversity of SBCs (Lu et al., 2022).

In some small-scale habitats, light may be a key factor driving bacterial community aggregation (Li C. et al., 2023; Li X. et al., 2023). The correlation between the relative abundance of Proteobacteria and light intensity was significant in the EP sample plot. Shading by PV panels resulted in a reduction of its relative abundance, which contradicts the findings of another PV site (Bai et al., 2022). This difference may be attributed to variations in environmental factors and vegetation types in PV sites. The Mantel test and correlation analysis results showed soil nutrients were the key drivers of changes in soil dominant communities, while light had a less direct role in changes in soil dominant bacterial communities, and it indirectly shaped the microbial community through its effects on the plant community (Li C. et al., 2023). The two-factor correlation network analysis revealed that the core SBCs in the *EP* sample plot were mainly negatively correlated with soil nutrients, while the core soil bacteria species in the *LC* sample plot were positively correlated with soil nutrients. This suggests that the different types of vegetation restoration have significant impacts on the soil environment and subsurface micro-ecosystems.

## 5 Conclusion

This study used high-throughput sequencing technology to investigate the effects of light gradient and soil depth on the composition and diversity of SBCs. It also explored survival strategies and key drivers of SBCs under two vegetation restoration modes. The results showed significant differences in light intensity among different locations (*p* < 0.05) with the order as follows: CK > FP > RP > UP. PV shading led to a 20–30% increase in soil microbial biomass. However, the OTU numbers, Shannon diversity, ACE, and Chao1 richness indices all decreased to varying degrees. The sampling depth had a greater effect on the diversity and relative abundance of SBCs than light intensity. The five most dominant phyla were Actinobacteria, Proteobacteria, Acidobacteria, Chlorobacteria, and Dimonobacteria. Species with a relative abundance of less than 0.01% were significantly more affected by light intensity than those with a high relative abundance. Network analysis revealed that symbiotic relationships dominated critical SBCs in the *LC* sample plot, whereas competitive interactions increased significantly in the *EP* sample plot, meaning that the ecological network was more stable and better adapted to light stress in the PV plots. *EP* is more conducive to maintaining the stability and health of subsurface ecosystems in vulnerable areas compared to *LC*. Soil nutrients are the key driving factor for changes in the dominant soil community.

## Data availability statement

The datasets presented in this study can be found in online repositories. The names of the repository/repositories and accession number(s) can be found at: https://www.ncbi.nlm.nih.gov/, PRJNA913967.

## Author contributions

ZL: Conceptualization, Data curation, Formal analysis, Investigation, Methodology, Writing – original draft, Writing – review & editing. JL: Data curation, Investigation, Writing – review & editing. SW: Investigation, Writing – review & editing. XL: Investigation, Writing – review & editing. XS: Conceptualization, Funding acquisition, Resources, Supervision, Writing – review & editing. All authors contributed to the article and approved the submitted version.

## Footnotes

^1^ http://drive5.com/uparse/
