# Supplementary material for: Soil bacterial community in a photovoltaic system adopted different survival strategies to cope with small-scale light stress under different vegetation restoration modes
Source: Front Microbiol. 2024 Mar 14;15:1365234. doi: 10.3389/fmicb.2024.1365234 (PMC10972850; doi:10.3389/fmicb.2024.1365234)
Supplement: Supplementary file 1 [file Table_1.docx]

**Supplementary material**

**of**

**Soil bacterial community in a photovoltaic system adopted different survival strategies** **to cope with small-scale light stress** **under different** **vegetation restoration modes**

**Zhongxin Luo^1,2^, Jiufu Luo^1,2^,** **Sainan Wu^1,2^, Xiaolin Luo^1,2^, Xin Sui^1,2*^**

^1^China Institute of Water Resources and Hydropower Research, Beijing 100038, China

^2^National Research Center for Sustainable Hydropower Development, Beijing 100038, China

***Correspondence:**
Xin Sui

suixin@iwhr.com


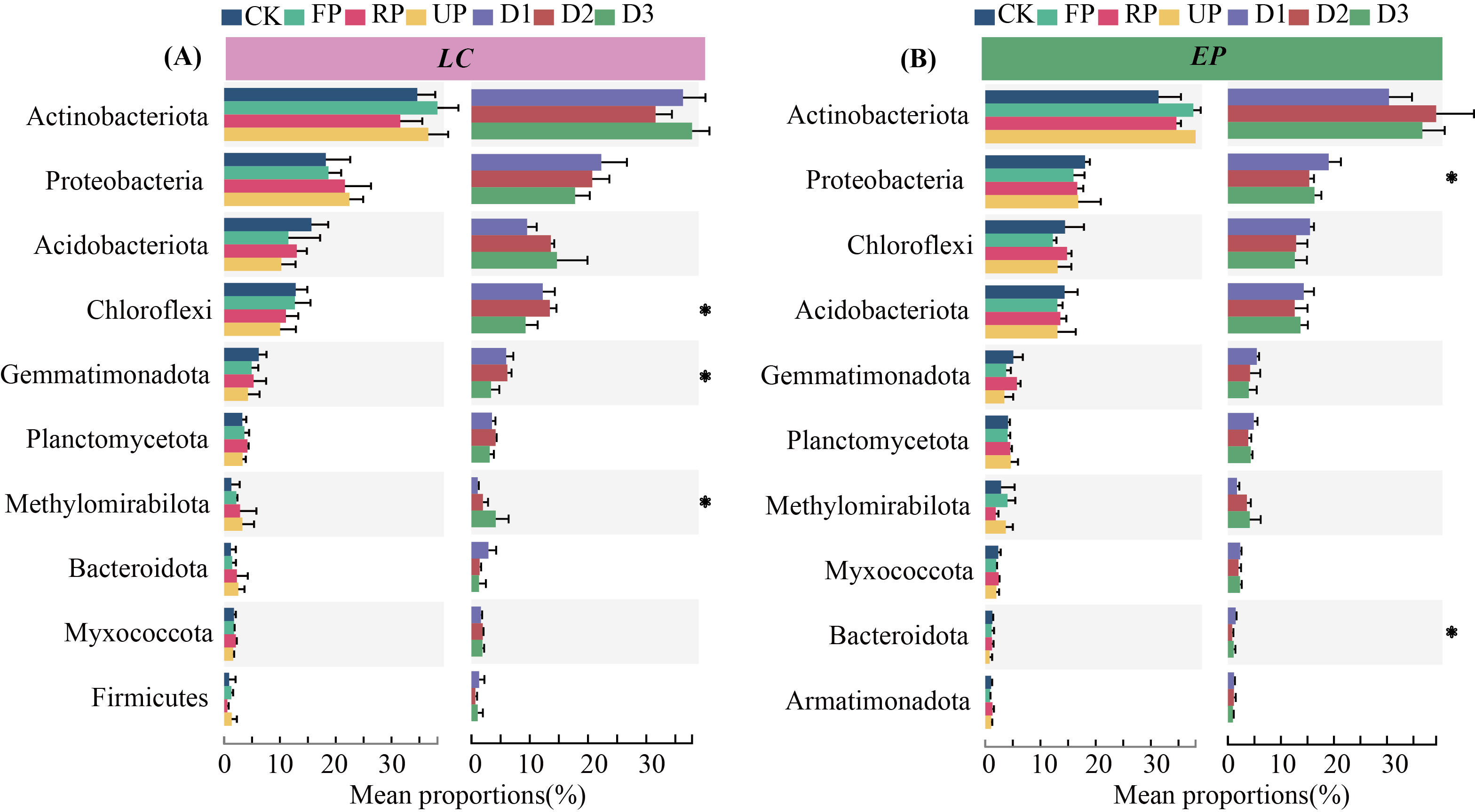


Fig. S1 Kruskal-Wallis H test bar plot along light gradient and sampling depth in two sample plots at phylum level (top 10): (A) *LC*; (B) *EP*


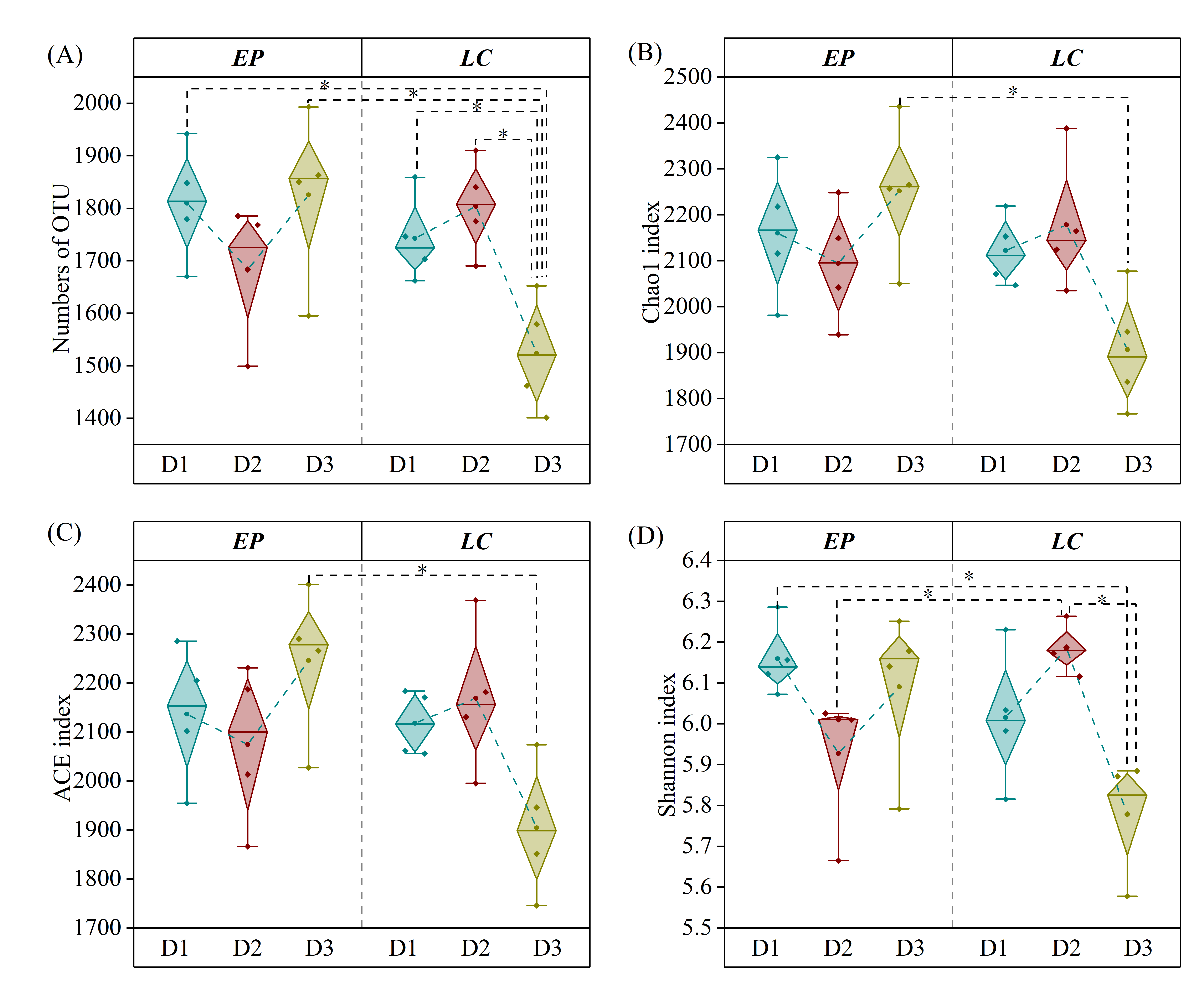


Fig. S2 Alpha diversity analysis of SBCs in different soil depths in two vegetation sample plots: (A) numbers of OTU, (B) Chao1 richness index, (C) ACE evenness index, and (D) Shannon diversity index

Table S1 Soil properties in the PV field under vegetation restoration modes

| Properties | *EP* | | | | | | *LC* | | | | |
| --- | --- | --- | --- | --- | --- | --- | --- | --- | --- | --- | --- |
|  | CK | FP | RP | UP | Mean | CK | | FP | RP | UP | Mean |
| TN(g/kg) | 1.05±0.22ab | 0.85±0.22ab | 1.18±0.03a | 0.97±0.26ab | 1.01 | 0.9±0ab | | 1±0.17ab | 0.82±0.13b | 0.77±0.12b | 0.87 |
| TC(g/kg) | 6.6±2.21a | 4.85±2.66a | 7.88±0.1a | 6.22±3.74a | 6.39 | 5.42±0.44a | | 5.42±1.52a | 4.9±1.96a | 4.43±0.98a | 5.04 |
| NO_3_^-^-N(mg/kg) | 23.00±1.33a | 15.30±7.1abc | 21.78±5.24ab | 9.24±4.7bc | 17.33 | 14.51±9.14abc | | 18.75±10.42abc | 6.74±3.11c | 6.42±3.05c | 11.61 |
| WSOC(mg/kg) | 102.17±40.93a | 101.57±42.26a | 97.33±26.05a | 97.33±37.55a | 99.60 | 74.67±4.83a | | 123.22±90.77a | 103.45±31.93a | 81.82±18.3a | 95.79 |
| MBC(mg/kg) | 196.24±31.52ab | 212.12±62.75a | 230.23±32.38a | 211.83±18.74a | 212.60 | 129.62±15.23b | | 240.78±30.06a | 225.15±22.93a | 248.1±51.39a | 210.91 |
| AK(mg/kg) | 281.34±168.01a | 225.13±83.98a | 405.25±122.24a | 218.79±77.46a | 282.63 | 280.3±65.98a | | 344.1±112.48a | 298.99±86.94a | 293.28±115.42a | 304.17 |
| AP (mg/kg) | 2.38±1.19a | 2.06±0.71a | 2.29±0.49a | 2.69±1.58a | 2.36 | 2.68±1.24a | | 2.16±0.82a | 1.95±0.3a | 1.66±0.69a | 2.12 |
| pH | 7.24±0.08a | 7.23±0.12a | 7.12±0.14a | 7.23±0.24a | 7.21 | 7.1±0.05a | | 7.14±0.09a | 7.02±0.15a | 7.39±0.31a | 7.16 |
| Humidity (%) | 14.85±1.79d | 17.62±1.3c | 13.53±0.62d | 18.02±0.63bc | 16.00 | 19.13±1.5abc | | 21.02±0.42a | 19.97±1.26ab | 21.25±0.48a | 20.34 |
| EC(μs/cm) | 220.6±37.6a | 185.23±40.72a | 341.53±145.85a | 231.53±55.19a | 244.73 | 240.57±116.62a | | 341.17±307.5a | 181.67±48.47a | 154.53±14a | 229.48 |

Notes: Values are means ± standard deviation. Different lowercase letters indicate a significant difference among different locations for two vegetation types (one-way ANOVA, p<0.05). TC, total carbon; TN, total nitrogen; NO_3_^-^-N, nitrate nitrogen; WSOC, water-soluble organic carbon; MBC, microbial carbon; AK, available potassium; AP, available phosphorus; EC, electric conductivity
